# Top food categories contributing to Canadian children’s energy and nutrient intakes at school

**DOI:** 10.1371/journal.pone.0340494

**Published:** 2026-01-13

**Authors:** Emily R. Ziraldo, Emma Wedekind, Mavra Ahmed, Daniel W. Sellen, Mary R. L’Abbé

**Affiliations:** 1 Department of Nutritional Sciences, Temerty Faculty of Medicine, University of Toronto, Toronto, Ontario, Canada; 2 Dalla Lana School of Public Health, University of Toronto, Toronto, Ontario, Canada; 3 Joannah & Brian Lawson Centre for Child Nutrition, University of Toronto, Toronto, Ontario, Canada; 4 Department of Anthropology, University of Toronto, Toronto, Ontario, Canada; University of Georgia, UNITED STATES OF AMERICA

## Abstract

**Introduction:**

Understanding the foods and beverages that children consume at school can help inform the development of school food programs, which has become a priority in Canada following the announcement of federal funding commitments and release of Canada’s first National School Food Policy in 2024. Therefore, the objective was to identify top sources of energy, nutrients to limit (sodium, sugars, and saturated fat), and nutrients to encourage (potassium, calcium, fibre, iron, and vitamin A) consumed by Canadian children at school.

**Methods:**

Intake data from the first day 24-hour recall of the 2015 Canadian Community Health Survey – Nutrition was examined for children 4–18 y (n = 1,690) who consumed food at school. Foods and beverages were grouped into 28 categories (e.g., baked goods, fruit and vegetable juice and drinks etc.). Top categories were identified by frequency of consumption and percent contribution to total population-level intakes.

**Results:**

Top sources of nutrients to limit often overlapped with top sources of nutrients to encourage. Handheld entrées was the top source of energy, sodium, saturated fat, calcium, and iron intakes at school. Fruits was the top source of sugars, potassium, and fibre and vegetables was the top source of vitamin A. By age group and sex, top sources were mostly similar with some differences in rankings and percent contributions.

**Conclusions:**

Results identified the top sources of energy, nutrients to limit, and nutrients to encourage consumed by Canadian children at school. Knowledge of these sources can inform the development of school food programs that aim to improve the diet quality of Canadian children at school.

## Introduction

Over the past three decades, childhood obesity rates in Canada have nearly tripled, putting children at risk for adverse health outcomes including type 2 diabetes, hypertension, cardiovascular disease, depression, low self-esteem, eating disorders, and social isolation [1,2]. Unhealthy diet is a major preventable risk factor for obesity and Canadian children have poor diet quality [3], failing to meet intake recommendations for fruits and vegetables and overconsuming processed foods that are high in sodium, sugars, and saturated fat [4,5].

Given that Canadian children often fail to meet dietary guidelines and consume on average one third of their daily calories during school hours [6], schools and school food programs have the potential play a pivotal role in shaping healthier eating habits. In the United States, evaluations of the federally assisted National School Lunch Program and School Breakfast Program suggest that school food programs improve diet quality, especially for students who consume lower quality diets, have limited access to food and/or are nutritionally disadvantaged at home [7]. Canada has had a patchwork of school food programs, mostly in elementary schools, that are varied in their scope, resources, and funding sources [8,9], however, there has been recent investment in Canada’s first national school food program. In April 2024, the federal government announced funding for a new National School Food Program [10], in June 2024 the National School Food Policy was published [11], with all provinces signing agreements to participate by March 2025 [12], and in October 2025 it was announced that the federal government would take steps to make the National School Food Program permanent [13].

Research investigating Canadian children’s food consumption at school has assessed nutrient intakes, broad food groups, and dietary quality [6,14–19], however, there have been no studies that identify food categories Canadian children consume frequently at school. Understanding what foods are consumed at school and how they contribute to energy and nutrient intakes can inform school food program development by highlighting foods where children’s nutrient intakes can be improved with the greatest impact. Therefore, the objective was to identify the top sources of energy, nutrients to limit (i.e., sodium, sugars, and saturated fat), and nutrients to encourage (i.e., potassium, calcium, fibre, iron, and vitamin A) consumed by Canadian children at school overall, by age group, and by sex.

## Methods

### Data source and study population

Dietary intakes of Canadian school-age children, 4–18 y, were assessed using the 2015 Canadian Community Health Survey (CCHS)-Nutrition Public Use Microdata Files [20]. The 2015 CCHS-Nutrition was conducted by Statistics Canada and is the most recent, nationally representative health and nutrition survey in Canada. Statistics Canada granted ethical approval for the survey data collection, which was collected under the authority of the Statistics Act of Canada, with informed consent obtained for all participants. Additionally, the University of Toronto Research Ethics Board does not require review for secondary analysis of anonymous human data. Individuals aged 1 year and older living in private dwellings in the 10 Canadian provinces were included and sampling was planned to capture a minimum number of individuals in each dietary reference intake age and sex group [21,22]. The 2015 CCHS-Nutrition included two components: 1) a 24-hour dietary recall (24HR) and 2) a general health questionnaire to obtain sociodemographic, anthropometric, health status, and lifestyle data. For children <6 y, parents completed the interview and for children 6–11 y, the interview was conducted by the child with parent assistance. Interviews were conducted between January 2, 2015 and December 31, 2015 and the data was accessed for this research in January 2022. The Public Use Microdata Files include all individual respondent records but certain variables were excluded or altered to protect respondent confidentiality.

To enable estimation of energy and nutrient intakes, foods and beverages reported in the 24HRs were linked to nutrition information in the 2015 Canadian Nutrient File [23], Health Canada’s standard food composition database, and a recipe database using the Nutrition Survey System codes [21]. A subset of 2015 CCHS-Nutrition participants (35%) completed a second 24HR between 3 and 10 days after the first 24HR, which was completed by all participants [21]. Due to day-to-day variation in food consumption, data from single-day 24HRs are affected by within-person variation and do not accurately represent an individual’s usual intake [24]. The administration of a second 24HR enables adjustment for within-person variation in intakes and therefore, usual intake distributions can be estimated. This analysis considered intakes at the population level and, as mean intake from a single 24HR is an acceptable estimate of mean usual intake of a group [24], only data from the first 24HR was used, as has been done previously for Canadian children and adults [25] and American children [26].

Out of 20,487 respondents in the 2015 CCHS-Nutrition, individuals were excluded if they were <4 or >18 y (n = 15,243), as Canadian children usually start elementary school at 4 y and complete secondary school at 18 y [27], were breastfeeding (n = 1), completed their 24HR for a weekend day (n = 2,022), or did not consume any foods or beverages at school (n = 1,531). The final sample included 1,690 participants. An exclusions flow chart, sub-analysis to investigate why 1,531 children aged 4–18 y did not consume food at school on a weekday, and sociodemographic characteristics of the final analytic sample can be found elsewhere [28].

### Categorization of foods and beverages

Using the Nutrition Survey System codes (n = 1,037 unique codes), all foods and beverages (n = 7,408) consumed by participants at school were categorized into 28 mutually exclusive categories (Table 1). Foods and beverages consumed as part of a recipe were categorized at the main recipe level using the Nutrition Survey System codes for the main recipe. Foods and beverages consumed as ingredients were categorized at the food level using the Nutrition Survey System food codes. Food categories were informed by established federal and provincial categorizations systems, specifically Health Canada’s Table of Reference Amounts for food [29] and the Government of Ontario’s School Food and Beverage Policy [30].

**Table 1:** Food categories (n = unique Nutrition Survey System codes within each category) used for assessment of top sources of Canadian children’s energy and nutrient intakes at school.

Babyfood	6
Babyfood	6
**Bakery products**	**174**
Baked goods (e.g., muffins, cookies, granola bars, energy bars, protein bars, croissants, pastries, pies, cakes, donuts)	105
Bread products (e.g., breads, buns, bagels, biscuits, English muffins, pitas, tortillas, bannock)	44
Breakfast cereals and granola	25
**Beverages**	**99**
Coffee, tea, and hot chocolate	15
Fruit and vegetable juice and drinks	48
Other beverages (e.g., soda, energy drinks, sports drinks, vitamin water, lemonade, flavoured water)	30
Water	6
**Combination dishes and entrées**	**290**
Combination dishes (e.g., shepherd’s pie, chicken with rice and vegetables, beef and noodles, meat pies, vegetable and meat lasagna, macaroni and cheese, and vegetarian and meat chili)	94
Handheld entrées (e.g., sandwiches, wraps, burgers, pizza, hotdogs, lunch kits, sushi)	119
Side dishes and hors d'oeuvres (e.g., plain pasta, noodles, rice, vegetable salads, potatoes, dumplings, samosas)	52
Soups and broth	25
**Confectionary and desserts**	**57**
Candies, chocolate, and desserts (e.g., pudding, gelatin, mousse)	57
**Dairy**	**56**
Cheese	22
Milk, yogurt drinks, and plant- based beverages	12
Yogurt	22
Fruits and vegetables	126
Fruits (includes fresh, frozen, cooked, bottled, canned, and dried)	61
Vegetables (includes fresh, frozen, cooked, bottled, canned, and dried)	65
**Meat, meat alternatives, eggs, and fish**	**109**
Eggs, tofu, legumes, and meat alternatives	13
Fish and seafood	6
Meat and poultry	74
Nuts, seeds, and nut butters	16
**Miscellaneous additions**	**64**
Cream, condensed milk, and non- dairy creamer	4
Fats and oils	7
Meal replacements	3
Sauces, dips, spreads, dressings, and condiments	37
Sweet toppings (e.g., syrups, honey, sugar, jams)	13
**Snacks**	**56**
Snack foods (e.g., chips, crackers, crispbread, popcorn)	56

### Statistical analysis

This study focused on energy, three nutrients to limit (i.e., sodium, sugars, and saturated fat) and five nutrients to encourage (i.e., calcium, potassium, fibre, iron, and vitamin A) for Canadian children. Nutrients to limit were identified based on recommendations from food policies under Health Canada’s Healthy Eating Strategy that identify sodium, sugars, and saturated fat as nutrients for which excess intake should be avoided [31]. Nutrients to encourage were identified as having a high prevalence of inadequacy among all school-age children in Canada, or for specific groups (i.e., 14–18 y females have a high prevalence inadequate iron intakes) [32].

The frequency of consumption and percent contribution to total intakes of energy and nutrients were reported by food category. The percent contribution was calculated at the population level by summing the total amount of a dietary component consumed at school from a food category and dividing by the total amount of the dietary component consumed from all categories across all children at school (i.e., per capita), as shown in Eq.1:

(1) Population ratio formula

$${Population\;ratio}_{j}=\frac{\sum_{i=\text{1}}^{n}E_{i,j}\cdot W_{i}}{\sum_{i=\text{1}}^{n}E_{i}\cdot W_{i}}\cdot \text{1}\text{0}\text{0}$$

where *n* is the total number of participants, *E*_*i,j*_ is the total intake of a dietary component (e.g., energy) for each participant from a food category (e.g., fruits), *W*_*i*_ is the survey weight for each participant, and *E*_*i*_ is the total intake of a dietary component (e.g., energy) consumed by all participants across all food categories. This approach, known as the population ratio method, is consistent with previous studies investigating the top sources of dietary components [25,26] and is preferred over calculating the mean of each individual’s ratio because it is more representative of usual intake [33]. For each food category, the mean amount of each dietary component consumed was calculated both per capita and per consumer of the food category. To account for the complex survey design, bootstrapping with 500 replicates was used to generate standard error estimates for each mean. The sample weights provided by Statistics Canada were applied to obtain estimates generalizable to the Canadian population and results were reported for the overall sample, by age group, and by sex. Age groups were divided as younger children aged 4–9 y and adolescents aged 10–18 y, based on World Health Organization’s definition of adolescence [34]. Detailed methodology for the calculation of the sampling weights is described in the 2015 CCHS-Nutrition Public Use Microdata Files User Guide [22]. As recommended by Statistics Canada, any estimates with small sample sizes (n < 10) or an unacceptably high coefficient of variation (>33.3) were considered not reliable and were not published, while estimates with a coefficient of variation >16.6 and ≤33.3 were flagged for high sampling variability [22]. Data manipulation and analysis was conducted using R version 4.2.1.

## Results

### Frequency of food source consumption

The 10 most frequently consumed food categories among children at school were identified (Table 2). Among all children, the five most frequently consumed categories were water (consumed by 64.8% of children), fruits (55.2%), baked goods (45.4%), handheld entrées (44.7%), and fruit and vegetable juice and drinks (35.7%). By age group, the top six most frequently consumed categories were the same as those among all children, with slight variations in rankings. Fruits were consumed more frequently by younger children (by 67.7%) than adolescents (45.2%). Cheese and milk, yogurt drinks and plant-based beverages were not in the top 10 most frequently consumed categories for adolescents but were for younger children (#8, #9), males (#9, #8), and females (#8, #10).

**Table 2:** Top 10 food categories consumed most frequently by 4-18 year old children at school by age group, sex, and among all children, ranked by weighted proportion of consumers, 2015 CCHS-Nutrition – Public Use Microdata Files (n = 1,690).

	Top 10 food categories* (%)				
	Age Group		Sex		Overall
	Younger children (i.e., 4–9 y; n = 575)	Adolescents (i.e., 10–18 y; n = 1,115)	Males (n = 842)	Females (n = 848)	All children (n = 1,690)
1	Water (68.7)	Water (61.7)	Water (66.9)	Water (62.7)	Water (64.8)
2	Fruits (67.7)	Fruits (45.2)	Fruits (54.0)	Fruits (56.3)	Fruits (55.2)
3	Baked goods (50.6)	Handheld entrées (42.9)	Baked goods (51.3)	Handheld entrées (40.6)	Baked goods (45.4)
4	Handheld entrées (46.8)	Baked goods (41.3)	Handheld entrées (48.7)	Baked goods (39.5)	Handheld entrées (44.7)
5	Fruit and vegetable juice and drinks (43.6)	Fruit and vegetable juice and drinks (29.4)	Fruit and vegetable juice and drinks (35.5)	Fruit and vegetable juice and drinks (35.9)	Fruit and vegetable juice and drinks (35.7)
6	Snack foods (36.5)	Snack foods (22.0)	Snack foods (25.8)	Snack foods (31.0)	Snack foods (28.4)
7	Vegetables (29.7)	Combination dishes (16.4)	Vegetables (20.1)	Vegetables (24.0)	Vegetables (22.0)
8	Cheese (26.0)	Vegetables (15.9)	Milk, yogurt drinks, and plant-based beverages (16.0)	Cheese (19.1)	Cheese (17.5)
9	Milk, yogurt drinks, and plant-based beverages (24.6)	Side dishes and hors d’oeuvres (12.5)	Cheese (15.9)	Yogurt (17.6)	Milk, yogurt drinks, and plant-based beverages (16.8)
10	Yogurt (20.7)	Yogurt (11.6)	Combination dishes (13.8^†^)	Milk, yogurt drinks, and plant-based beverages (17.5)	Yogurt (15.6)

Note: Percents represent the weighted proportion of children who consumed each food category at school.

Abbreviation: CCHS, Canadian Community Health Survey.

*Examples of foods in categories include: **Baked goods** such as muffins, cookies, granola bars, energy bars, protein bars, croissants, pastries, pies, cakes, and donuts; **combination dishes** including shepherd’s pie, chicken with rice and vegetables, beef and noodles, meat pies, vegetable and meat lasagna, macaroni and cheese, and vegetarian and meat chili; **handheld entrées** like sandwiches, wraps, burgers, pizza, hotdogs, lunch kits, and sushi; **fruits** including all fresh, frozen, cooked, bottled, canned, and dried fruit; **side dishes and hors d’oeuvres** like plain pasta, noodles, rice, vegetable salads, potatoes, dumplings, and samosas; **snack foods** such as chips, crackers, crispbread, and popcorn; and **vegetables** including all fresh, frozen, cooked, bottled, canned, and dried vegetables. See Table 1 for complete list of categories and details of the components of each category.

†The coefficient of variation (CV) for this estimate has high sampling variability (i.e., 16.6 > CV ≤ 33.3).

### Top sources of energy and nutrients to limit for children at school

The results show the top three sources of energy and nutrients to limit at school for all children and stratified by age group and sex (Table 3). Among all children, handheld entrées were the top source (>11% higher compared to the second highest source) of energy (25.6% of intakes at school), sodium (44.9%), and saturated fat (33.3%). Fruits (24.7%), followed by fruit and vegetable juice and drinks (20.9%), were the top two sources of sugar intakes. The top 10 sources of children’s energy, sodium, sugars, and saturated fat intakes at school contributed 86.7%, 91.6%, 92.9%, and 90.9% of intakes, respectively (S1–S4 Tables). The mean amounts consumed (both per capita and per consumer), as well as the n (%) of children consuming the top 10 food sources of energy, sodium, sugars, and saturated fat, are also detailed in S1–S4 Tables.

**Table 3:** Top three food categories contributing to intakes of energy and nutrients to limit consumed by 4-18 year old children at school by age group, sex, and among all children, ranked by proportion contributed, 2015 CCHS-Nutrition – Public Use Microdata Files (n = 1,690).

	Top 3 food categories* (%)				
	Age Group		Sex		Overall
	Younger children (i.e., 4–9 y; n = 575)	Adolescents (i.e., 10–18 y; n = 1,115)	Males (n = 842)	Females (n = 848)	All children (n = 1,690)
Energy					
1	Handheld entrées (22.7)	Handheld entrées (27.8)	Handheld entrées (27.4)	Handheld entrées (23.6)	Handheld entrées (25.6)
2	Baked goods (14.5)	Baked goods (13.2)	Baked goods (16.1)	Baked goods (11.1)	Baked goods (13.7)
3	Fruits (10.5)	Combination dishes (11.2^†^)	Combination dishes (9.3^†^)	Combination dishes (10.2)	Combination dishes (9.7)
Sodium					
1	Handheld entrées (40.3)	Handheld entrées (48.4)	Handheld entrées (47.8)	Handheld entrées (41.6)	Handheld entrées (44.9)
2	Combination dishes (9.2^†^)	Combination dishes (13.7^†^)	Combination dishes (11.5^†^)	Combination dishes (12.1)	Combination dishes (11.8)
3	Snack foods (8.7)	Snack foods (7.2^†^)	Baked goods (8.4)	Snack foods (9.2)	Snack foods (7.8)
Sugars					
1	Fruits (28.3)	Fruits (21.5)	Fruits (23.7)	Fruits (25.7)	Fruits (24.7)
2	Fruit and vegetable juice and drinks (20.6)	Fruit and vegetable juice and drinks (21.2)	Fruit and vegetable juice and drinks (19.8)	Fruit and vegetable juice and drinks (22.1)	Fruit and vegetable juice and drinks (20.9)
3	Baked goods (14.7)	Baked goods (16.6)	Baked goods (18.7)	Baked goods (12.5)	Baked goods (15.7)
Saturated fat					
1	Handheld entrées (25.2)	Handheld entrées (39.2^†^)	Handheld entrées (33.0)	Handheld entrées (33.6^†^)	Handheld entrées (33.3)
2	Baked goods (16.5)	Baked goods (12.7)	Baked goods (18.2)	Cheese (11.1^†^)	Baked goods (14.3)
3	Cheese (16.1^†^)	Combination dishes (12.5^†^)	Combination dishes (9.7^†^)	Baked goods (10.3)	Cheese (10.3)

Note: Percents represent the weighted average proportion of each dietary component contributed by food category. S1–S4 Tables summarize the top 10 categories contributing to intakes of energy and nutrients to limit overall and the top three categories by age and sex group, including the weighted mean amount contributed from each category per capita, the weighted mean amount contributed from each category per consumer, and the number and weighted proportion of consumers.

Abbreviation: CCHS, Canadian Community Health Survey.

*Examples of foods in categories include: **Baked goods** such as muffins, cookies, granola bars, energy bars, protein bars, croissants, pastries, pies, cakes, and donuts; **bread products** like breads, buns, bagels, biscuits, English muffins, pitas, tortillas, and bannock; **combination dishes** including shepherd’s pie, chicken with rice and vegetables, beef and noodles, meat pies, vegetable and meat lasagna, macaroni and cheese, and vegetarian and meat chili; **handheld entrées** like sandwiches, wraps, burgers, pizza, hotdogs, lunch kits, and sushi; **fruits** including all fresh, frozen, cooked, bottled, canned, and dried fruit; and **snack foods** such as chips, crackers, crispbread, and popcorn. See Table 1 for complete list of categories and details of the components of each category.

†The coefficient of variation (CV) for this estimate has high sampling variability (i.e., 16.6 > CV ≤ 33.3).

The top three sources of energy among all children were handheld entrées (25.6%), baked goods (13.7%), and combination dishes (9.7%). For both age groups and sexes, handheld entrées and baked goods were the top two sources of energy, however, the third largest source was fruits for younger children and combination dishes for adolescents, males and females.

For sodium, the top three sources among all children were handheld entrées (44.9%), combination dishes (11.8%), and snack foods (7.8%). Handheld entrées and combination dishes were the top two sources of sodium for both age groups and sexes, however, the third largest source was baked goods for males and snack foods for females, younger children, and adolescents.

For sugars, the top three sources among all children were fruits (24.7%), fruit and vegetable juice and drinks (20.9%), and baked goods (15.7%). The top three sources were the same across age groups and sexes.

For saturated fat, the top three sources among all children were handheld entrées (33.3%), baked goods (14.3%), and cheese (10.3%). Handheld entrées and baked goods were in the top three sources of saturated fat across both age groups and sexes. By age group, cheese was the third largest source for younger children while combination dishes was the third largest source for adolescents. By sex, combination dishes was the third largest source for males while cheese was the second largest source for females.

### Top sources of nutrients to encourage for children at school

The results show the top three sources of nutrients to encourage at school for all children and stratified by age group and sex (Table 4). Handheld entrées was the top source of calcium (26.5%) and iron (31.8%) intakes. Fruits was the top source of potassium (19.6%) and fibre (27.7%). Vegetables was the top source of vitamin A (24.9%). The top 10 sources of nutrients to encourage contributed 88.4%, 89.3%, 94.5%, 90.8%, and 90.8% of children’s calcium, potassium, fibre, iron, and vitamin A intakes at school, respectively (S5–S9 Tables). The mean amounts consumed (both per capita and per consumer), as well as the n (%) of children consuming the top 10 food sources of calcium, potassium, fibre, iron, and vitamin A, are also detailed in S5–S9 Tables.

**Table 4:** Top three food categories contributing to intakes of energy and nutrients to encourage consumed by 4-18 year old children at school by age group, sex, and among all children, ranked by proportion contributed, 2015 CCHS-Nutrition – Public Use Microdata Files (n = 1,690).

	Top 3 food categories* (%)				
	Age Group		Sex		Overall
	Younger children (i.e., 4–9 y; n = 575)	Adolescents (i.e., 10–18 y; n = 1,115)	Males (n = 842)	Females (n = 848)	All children (n = 1,690)
Calcium					
1	Milk, yogurt drinks, and plant-based beverages (22.2^†^)	Handheld entrées (31.9)	Handheld entrées (29.9)	Handheld entrées (23.3)	Handheld entrées (26.5)
2	Handheld entrées (20.5)	Milk, yogurt drinks, and plant-based beverages (11.2)	Milk, yogurt drinks, and plant-based beverages (14.8)	Milk, yogurt drinks, and plant-based beverages (18.0^†^)	Milk, yogurt drinks, and plant-based beverages (16.4)
3	Cheese (17.4)	Baked goods (8.4)	Cheese (12.1)	Cheese (12.9^†^)	Cheese (12.5)
Potassium					
1	Fruits (23.9)	Handheld entrées (19.3)	Handheld entrées (18.7)	Fruits (20.7)	Fruits (19.6)
2	Handheld entrées (14.0)	Fruits (16.1)	Fruits (18.4)	Handheld entrées (15.2)	Handheld entrées (16.9)
3	Fruit and vegetable juice and drinks (13.9)	Fruit and vegetable juice and drinks (13.9)	Fruit and vegetable juice and drinks (14.1)	Fruit and vegetable juice and drinks (13.6)	Fruit and vegetable juice and drinks (13.9)
Fibre					
1	Fruits (32.7)	Handheld entrées (26.5)	Fruits (27.8)	Fruits (27.6)	Fruits (27.7)
2	Handheld entrées (22.1)	Fruits (23.6)	Handheld entrées (25.6)	Handheld entrées (23.3)	Handheld entrées (24.5)
3	Baked goods (9.3)	Baked goods (13.0)	Baked goods (13.9)	Combination dishes (9.1^†^)	Baked goods (11.3)
Iron					
1	Handheld entrées (29.1)	Handheld entrées (33.7)	Handheld entrées (35.5)	Handheld entrées (28.0)	Handheld entrées (31.8)
2	Baked goods (17.2)	Baked goods (15.0)	Baked goods (19.2)	Baked goods (12.6)	Baked goods (15.9)
3	Combination dishes (10.0^†^)	Combination dishes (12.5^†^)	Combination dishes (11.3^†^)	Combination dishes (11.5^†^)	Combination dishes (11.4^†^)
Vitamin A					
1	Vegetables (29.3)	Vegetables (20.5^†^)	Vegetables (20.5)	Vegetables (28.2)	Vegetables (24.9)
2	Milk, yogurt drinks, and plant-based beverages (16.1^†^)	Handheld entrées (16.7^†^)	Handheld entrées (16.8)	Milk, yogurt drinks, and plant-based beverages (13.0^†^)	Handheld entrées (13.5)
3	Handheld entrées (10.3^†^)	Combination dishes (11.6^†^)	Milk, yogurt drinks, and plant-based beverages (12.4^†^)	Handheld entrées (11.1^†^)	Milk, yogurt drinks, and plant-based beverages (12.8)

Note: Percents represent the weighted average proportion of each dietary component contributed by food category. S5–S9 Tables summarize the top 10 categories contributing to intakes of energy and nutrients to encourage overall and the top three categories by age and sex group, including the weighted mean amount contributed from each category per capita, the weighted mean amount contributed from each category per consumer, and the number and weighted proportion of consumers.

Abbreviation: CCHS, Canadian Community Health Survey.

*Examples of foods in categories include: **Baked goods** such as muffins, cookies, granola bars, energy bars, protein bars, croissants, pastries, pies, cakes, and donuts; **bread products** like breads, buns, bagels, biscuits, English muffins, pitas, tortillas, and bannock; **combination dishes** including shepherd’s pie, chicken with rice and vegetables, beef and noodles, meat pies, vegetable and meat lasagna, macaroni and cheese, and vegetarian and meat chili; **handheld entrées** like sandwiches, wraps, burgers, pizza, hotdogs, lunch kits, and sushi; **other beverages** such as soda, energy drinks, sports drinks, vitamin water, lemonade, and flavored water; **fruits** including all fresh, frozen, cooked, bottled, canned, and dried fruit; **vegetables** including all fresh, frozen, cooked, bottled, canned, and dried vegetables. See Table 1 for complete list of categories and details of the components of each category.

†The coefficient of variation (CV) for this estimate has high sampling variability (i.e., 16.6 > CV ≤ 33.3).

The top three sources of calcium at school among all children were handheld entrées (26.5%), milk, yogurt drinks, and plant-based beverages (16.4%), and cheese (12.5%). Handheld entrées and milk, yogurt drinks, and plant-based beverages were top two sources of calcium across both age groups and sexes, however, the third largest source was baked goods for adolescents and cheese for younger children, males and females. By age group, handheld entrées contributed a higher proportion of calcium intakes among adolescents (31.9%) than among younger children (20.5%) and milk, yogurt drinks, and plant-based beverages contributed a higher proportion of calcium intakes among children (22.2%) than among adolescents (11.2%).

For potassium, the top three sources among all children at school were fruits (19.6%), handheld entrées (16.9%), and Fruit and vegetable juice and drinks (13.9%). The top three sources of potassium were the same for both age groups and sexes.

The top three sources of fibre among all children at school were fruits (27.7%), handheld entrées (24.5%), and baked goods (11.3%). Fruits and handheld entrées were the top two sources of fibre for both age groups and sexes, however, the third largest source was combination dishes for females and baked goods for younger children, adolescents, and males.

The top three sources of iron among all children at school were handheld entrées (31.8%), baked goods (15.9%), and combination dishes (11.4%). The top three sources of iron were the same and their percent contributions to intakes were similar across age groups and sexes.

For vitamin A, the top three sources at school among all children were vegetables (24.9%), handheld entrées (13.5%), and milk, yogurt drinks, and plant-based beverages (12.8%). Vegetables were the top source of vitamin A for both age groups and sexes. Milk, yogurt drinks and plant-based beverages was a top three source of vitamin A for younger children (#2), males (#3), and females (#2) but not for adolescents while combination dishes was only a top three source for adolescents (#3).

### Overlap between top sources of dietary components

Overlap was observed between the top five sources of children’s intakes of energy, nutrients to limit, and nutrients to encourage at school (Table 5). Handheld entrées were in the top five sources of energy and all nutrients. Baked goods were a top five source of energy, all nutrients to limit and three nutrients to encourage (calcium, fibre, and iron). Combination dishes were a top five source of energy, two nutrients to limit (sodium and saturated fat), and four nutrients to encourage (potassium, fibre, iron, and vitamin A). The top source of sugars, fruits, ranked in the top five sources for three nutrients to encourage (potassium, fibre, and vitamin A) and ranked low (i.e., not in the top 10) as a contributor to sodium and saturated fat intakes at school.

**Table 5:** Overlap between the top five food categories contributing to energy intakes and their ranked contribution to nutrient intakes among 4-18 year old children at school, 2015 CCHS-Nutrition – Public Use Microdata Files (n = 1,690).

	Dietary components and rank								
Food category*	Energy	Sodium	Sugars	Saturated fat	Calcium	Potassium	Fibre	Iron	Vitamin A
Handheld entrées	1	1	5	1	1	2	2	1	2
Baked goods	2	4	3	2	4	7	3	2	6
Combination dishes	3	2	9	4	7	4	4	3	4
Fruits	4	≥10	1	≥10	8	1	1	7	5
Snack foods	5	3	≥10	6	9	6	5	4	≥10

Note: Food categories were ranked by average proportion of each dietary component contributed. S1–S9 Tables summarize the top 10 categories contributing to energy and nutrient intakes and the top three categories by age and sex group, including the mean amount contributed from each category per capita, the mean amount contributed from each category per consumer, and the number and weighted proportion of consumers.

Abbreviation: CCHS, Canadian Community Health Survey.

*Examples of foods in categories include: **Baked goods** such as muffins, cookies, granola bars, energy bars, protein bars, croissants, pastries, pies, cakes, and donuts; **combination dishes** including shepherd’s pie, chicken with rice and vegetables, beef and noodles, meat pies, vegetable and meat lasagna, macaroni and cheese, and vegetarian and meat chili; **handheld entrées** like sandwiches, wraps, burgers, pizza, hotdogs, lunch kits, and sushi; **fruits** including all fresh, frozen, cooked, bottled, canned, and dried fruit; and **snack foods** such as chips, crackers, crispbread, and popcorn. See Table 1 for complete list of categories and details of the components of each category.

## Discussion

This study identified the top sources of energy, nutrients to limit (i.e., sodium, sugars, and saturated fat), and nutrients to encourage (i.e., potassium, calcium, fibre, iron, and vitamin A), as well as the amount each source contributed, consumed by a nationally representative sample of Canadian children at school. Findings reveal significant overlap between the top sources of nutrients to limit and nutrients to encourage, highlighting the dual role of some foods (e.g., handheld entrées and baked goods) in both meeting nutritional needs and contributing to excess intakes of nutrients to limit. Although top sources were mostly similar across age groups and sexes, there were some differences in frequency of consumption, ranking, and percent contribution which suggest variation in consumption patterns by age and sex at school.

The rankings should be considered in the context of the proportion contributed, as some categories are very similar in terms of contribution. For example, among top sources of sugars for adolescents, fruit and vegetable juice and drinks (ranked #2; 21.2%) contributed almost the same proportion as fruits (ranked #1; 21.5%). The percent contributed can also help understand differences between age groups and sexes. For example, handheld entrées are the top source of sodium intakes at school for both age groups and sexes, but they make up a higher proportion of sodium intakes for adolescents (48.4%) and males (47.8%) compared to younger children (40.3%) and females (41.6%). The frequency of consumption data (Table 2) and the mean intake per capita and per consumer (S1–S9 Tables) provide information to distinguish if a food is a top source because it is consumed often by a large proportion of children at school or if it is consumed by few children but contributing a large proportion of intakes due to its nutritional composition being high in a dietary component. For example, although vegetables was consumed by only 22.0% of children at school, it was the top source of vitamin A intakes (24.9%), likely due to the higher nutrient density of foods in the category, and consequently the mean intake of vitamin A from vegetables is much larger among consumers (180.7 ± 21.1 mcg RAE) than per capita (39.8 ± 5.2).

Previous research investigating food groups consumed by Canadian children during the school day has reported the number of servings of each food group consumed [6,14–17], however, data on the frequency of consumption of food sources is limited. A study conducted in Southern Ontario compared foods brought and consumed at school by elementary school students (grade 3–4, ages 7–10) across different eating schedules and found that, regardless of schedule, vegetable consumption was infrequent; only 40.8% of children had vegetables packed in their lunch and consumption was lower, with 20–30% of vegetables not consumed [19]. This is comparable with our finding that vegetables were consumed relatively infrequently, by 22.0%, of Canadian children at school.

Comparison of the results in this study against a comprehensive assessment of the top sources of energy, sodium, sugars, and saturated fat consumed by Canadian children (1–18 y) over the entire day [25] provides insight into how consumption patterns at school align with full day diets. Despite differences in food categories, mixed dishes appear as top sources of energy, sodium and saturated fat across the full day (e.g., meat-based mixed dishes, pizza, and pasta and pasta dishes) and at school (e.g., handheld entrées and combination dishes). Baked goods only appear as a top source at school but not over the full day, however, this may be due to grouping baked goods together in this study compared to leaving them in smaller categories in the assessment of full day intakes (e.g., cookies, cakes, granola and cereal bars). Milk intakes appear to differ over the full day compared to at school. For both 1–18 y males and 1–18 y females, unflavoured milk was a top source of energy (#2 for males and #1 for females, 6.8% and 6.6% of daily intakes), sugars (#2 for both, 12.1% and 11.5%), and saturated fat (#1 for both, 13.0% and 12.2%) over the entire day, while at school among 4–18 y, milk, yogurt drinks, and plant based beverages ranked lower – 8th for energy (3.9% of intakes at school), 4th for sugars (7.8%) and 7th for saturated fat (5.1%). This difference aligns with findings that Canadian children consume less milk and dairy products during school hours relative to non-school hours [6].

Our results also align more broadly with known dietary intake patterns of Canadian children. Canadian children consume diets high in nutrients to limit [4,35], which are often processed foods. Previous research has found that many of the food categories identified as frequently consumed by children at school in this study contain high proportions of processed foods [36]. For example, 100% of packaged bakery products were classified as processed or ultra-processed, which would include baked goods, the third most frequently consumed food category. Snack foods, the sixth most frequently consumed food category, are also often processed, with 98.8% of packaged snacks in Canada classified as processed or ultra-processed [36]. In 2017, 72% of Canadian children aged 4–13 years consumed amounts of sodium exceeding the recommended limits [37]. Health Canada has identified bakery products and mixed dishes as the top two food categories contributing to sodium intakes. In this analysis, many of the top ten sources of sodium intakes at school (i.e., handheld entrées, combination dishes, baked goods, bread products) fall under with these two broader food categories [37]. Despite the results alignment with known dietary intake patterns of Canadian children, it is important to take into consideration that the data were collected in 2015, which limits the contemporary relevance. The COVID-19 pandemic in 2020 forced school food programs to adapt and shift their focus to families with the greatest need [38,39] and changed food consumption patterns of children [40]. Additionally, the recent introduction of Canada’s first National School Food Policy in 2024, which invested $1 billion over five years to enhance and expand school food programs across all provinces and territories, may impact foods consumed at school in the years to come [12].

The National School Food Policy “articulates the federal government’s long-term vision for a national school food program in Canada” and presents an opportunity to improve the nutritional status and food-related behaviours of Canadian students [11]. One of the Policy objectives is to “help children meet their nutritional and health needs” by aligning foods with national and provincial or territorial guidelines [11]. Both Canada’s Food Guide and provincial and territorial guidelines, such as the Ontario School Food and Beverage Policy, recommend foods lower in sodium, sugars, and saturated fat [30,41]. Given this study found overlap between children’s top sources of nutrients to limit and nutrients to encourage, when possible, efforts to improve children’s nutrient intakes at school may focus on improving the nutritional composition of these sources. In mixed dishes that are top sources of nutrients to limit and encourage, like handheld entrées and combination dishes, nutritional composition may be improved by substituting ingredients typically high in nutrients to limit (e.g., white bread and deli meat) with ingredients that have a healthier nutrient profile (e.g., higher fibre whole grain bread or lower fat meat) and by adding nutrient-dense ingredients such as vegetables and fruits. For top sources of nutrients to limit that require less preparation or are often purchased pre-prepared, such as baked goods or fruit and vegetable juice and drinks, choosing options that are lower in sodium, sugars, and saturated fat or substituting for more nutrient dense alternatives, such as milk for fruit juice, may be a strategy to improve the nutrient intakes of children at school. However, the ability to substitute or add ingredients to improve the nutritional composition of foods consumed at school depends on many factors, including, but not limited to how, where, and by whom it is prepared, and should include consideration for children’s acceptance of the food.

Overall, this study supports a growing body of research to inform the development of school food programs by providing baseline data on foods children consume at school and how they contribute to intakes of energy, nutrients to limit, and nutrients to encourage. The results reflect the dietary intakes of Canadian children at school in 2015 and should not be interpreted as reflecting student food preferences, but rather, the outcome of the interplay of diverse factors influencing the intake of children at school. Examples of these factors include children’s food preferences, dietary patterns at the family or community level, access to school food programs, the school lunch environment, convenience or time, cost, allergy policies, and the cognitive, emotional and physical labour involved in the process of packing lunches for school [42–44]. Further research may investigate student and family food preferences at school, which is important to inform the development of school food programs that are acceptable to children and their families, and to explore differences in foods consumed at school by province, as diet quality at school [15], and school food program guidelines, food criteria, and participation rates vary by province [8].

### Strengths and limitations

Strengths of this research are the use of data from a large, nationally representative dietary intake survey with detailed dietary intake data from a 24HR and reporting by age groups and sex, which allow insight into consumption patterns of younger children versus adolescents and of males versus females at school.

There are limitations of this research. Results do not reflect consumption patterns of children who live on reserve, in remote areas, in the territories or in institutions, as the 2015 CCHS-Nutrition did not include these populations. Additionally, data on the quantity of salt added to foods during food preparation or at the time of food consumption was not collected and therefore, assessment and ranking of this source of sodium was not possible. For sugars, the 2015 CCHS-Nutrition and the Canadian Nutrient File [23] only report total sugars content of foods, so it was not possible to assess top sources of free or added sugars, which would be more informative for improving child nutrition as free and added sugars have a greater impact on health when consumed in excess. Additionally, although the 2015 CCHS-Nutrition did include a question about where each food was prepared, there was no option for if a food was prepared at school. As such, it was not possible to assess whether top sources of dietary components differed for foods prepared at school compared with those prepared at other locations (e.g., at home or in restaurants). This remains an interesting area for future research, as interventions to improve children’s nutrition at school will differ depending on where food is prepared.

This data only includes intakes at school and does not capture foods consumed off campus during the school day. Although most Canadian children consume lunch at school [16], it is possible that top sources of dietary components would be different for off-campus locations. This is expected to impact adolescents more than younger children, as older children more frequently consume lunch off campus [16].

Dietary intake data from 24HRs is known to be affected by misestimation of portion sizes [45], recall bias (e.g., respondents memory of food does not reflect what they consumed), and social desirability bias [46], which more frequently impacts the responses of younger children than older children and females than males [47]. To enhance accuracy, the 2015 CCHS-Nutrition used the Automated Multiple-Pass Method [48,49]. For children 4–6 y, parents completed the 24HR on behalf of their children, which may have introduced error if parents did not have an accurate estimation of how much food their child ate at school (e.g., if the child did not want to eat their lunch and discarded food at school). Finally, it is appropriate to use a single day 24HR to estimate mean usual intakes at the group level [24], as was done in this study. However, particularly for foods that are consumed infrequently, the proportion of consumers and the proportion contributed from each source may change if multiple days are considered as foods consumed at school vary from day-to-day.

## Conclusions

Knowledge of which foods are commonly consumed by Canadian children at school and how they contribute to energy and nutrient intakes is important to inform the development of school food programs that aim to improve children’s dietary quality. This study found overlap between top sources of nutrients to limit and nutrients to encourage and that most top sources were similar across age groups and sexes. The nutritional composition of school foods may be improved by offering healthier options (e.g., milk instead of fruit juice) or, by substituting ingredients in mixed dishes with healthier alternatives (e.g., whole grain instead of white bread in sandwiches).

## Supporting information

S1 Table Top food categories contributing to energy intakes of children at school, by age group, sex, and among all children, ranked by proportion contributed and including the mean amount per capita, the mean amount per consumer, and the number and proportion of children consuming each top category at school. 2015 CCHS-Nutrition – Public Use Microdata Files (n = 1,690). Note: The survey weights provided by Statistics Canada were applied to obtain nationally representative estimates. To account for the complex survey design, bootstrapping with 500 replicates was used to generate SE estimates. The counts (n’s) represent the unweighted number of children who consumed food in the category at school. Abbreviations: CCHS, Canadian Community Health Survey; SE, standard error. *Examples of foods in categories include: **Baked goods** such as muffins, cookies, granola bars, energy bars, protein bars, croissants, pastries, pies, cakes, and donuts; **bread products** like breads, buns, bagels, biscuits, English muffins, pitas, tortillas, and bannock; **combination dishes** including shepherd’s pie, chicken with rice and vegetables, beef and noodles, meat pies, vegetable and meat lasagna, macaroni and cheese, and vegetarian and meat chili; **handheld entrées** like sandwiches, wraps, burgers, pizza, hotdogs, lunch kits, and sushi; **fruits** including all fresh, frozen, cooked, bottled, canned, and dried fruit; **side dishes and hors d’oeuvres** like plain pasta, noodles, rice, vegetable salads, potatoes, dumplings, and samosas; and **snack foods** such as chips, crackers, crispbread, and popcorn. See Table 1 for complete list of categories and details of the components of each category. ^†^The coefficient of variation (CV) for this estimate has high sampling variability (i.e., 16.6 > CV ≤ 33.3).
(DOCX)

S2 Table Top food categories contributing to sodium intakes of children at school, by age group, sex, and among all children, ranked by proportion contributed and including the mean amount per capita, the mean amount per consumer, and the number and proportion of children consuming each top category at school. 2015 CCHS-Nutrition – Public Use Microdata Files (n = 1,690). Note: The survey weights provided by Statistics Canada were applied to obtain nationally representative estimates. To account for the complex survey design, bootstrapping with 500 replicates was used to generate SE estimates. The counts (n’s) represent the unweighted number of children who consumed food in the category at school. Abbreviations: CCHS, Canadian Community Health Survey; SE, standard error. *Examples of foods in categories include: **Baked goods** such as muffins, cookies, granola bars, energy bars, protein bars, croissants, pastries, pies, cakes, and donuts; **bread products** like breads, buns, bagels, biscuits, English muffins, pitas, tortillas, and bannock; **combination dishes** including shepherd’s pie, chicken with rice and vegetables, beef and noodles, meat pies, vegetable and meat lasagna, macaroni and cheese, and vegetarian and meat chili; **handheld entrées** like sandwiches, wraps, burgers, pizza, hotdogs, lunch kits, and sushi; **side dishes and hors d’oeuvres** like plain pasta, noodles, rice, vegetable salads, potatoes, dumplings, and samosas; and **snack foods** such as chips, crackers, crispbread, and popcorn. See Table 1 for complete list of categories and details of the components of each category. ^†^The coefficient of variation (CV) for this estimate has high sampling variability (i.e., 16.6 > CV ≤ 33.3).
(DOCX)

S3 Table Top food categories contributing to sugar intakes of children at school, by age group, sex, and among all children, ranked by proportion contributed and including the mean amount per capita, the mean amount per consumer, and the number and proportion of children consuming each top category at school. 2015 CCHS-Nutrition – Public Use Microdata Files (n = 1,690). Note: The survey weights provided by Statistics Canada were applied to obtain nationally representative estimates. To account for the complex survey design, bootstrapping with 500 replicates was used to generate SE estimates. The counts (n’s) represent the unweighted number of children who consumed food in the category at school. Abbreviations: CCHS, Canadian Community Health Survey; SE, standard error. *Examples of foods in categories include: **Baked goods** such as muffins, cookies, granola bars, energy bars, protein bars, croissants, pastries, pies, cakes, and donuts; **combination dishes** including shepherd’s pie, chicken with rice and vegetables, beef and noodles, meat pies, vegetable and meat lasagna, macaroni and cheese, and vegetarian and meat chili; **handheld entrées** like sandwiches, wraps, burgers, pizza, hotdogs, lunch kits, and sushi; **other beverages** such as soda, energy drinks, sports drinks, vitamin water, lemonade, and flavored water; and **fruits** including all fresh, frozen, cooked, bottled, canned, and dried fruit. See Table 1 for complete list of categories and details of the components of each category. ^†^The coefficient of variation (CV) for this estimate has high sampling variability (i.e., 16.6 > CV ≤ 33.3).
(DOCX)

S4 Table Top food categories contributing to saturated fat intakes of children at school, by age group, sex, and among all children, ranked by proportion contributed and including the mean amount per capita, the mean amount per consumer, and the number and proportion of children consuming each top category at school. 2015 CCHS-Nutrition – Public Use Microdata Files (n = 1,690). Note: The survey weights provided by Statistics Canada were applied to obtain nationally representative estimates. To account for the complex survey design, bootstrapping with 500 replicates was used to generate SE estimates. The counts (n’s) represent the unweighted number of children who consumed food in the category at school. Abbreviations: CCHS, Canadian Community Health Survey; NR, not reliable; SE, standard error. *Examples of foods in categories include: **Baked goods** such as muffins, cookies, granola bars, energy bars, protein bars, croissants, pastries, pies, cakes, and donuts; **combination dishes** including shepherd’s pie, chicken with rice and vegetables, beef and noodles, meat pies, vegetable and meat lasagna, macaroni and cheese, and vegetarian and meat chili; **handheld entrées** like sandwiches, wraps, burgers, pizza, hotdogs, lunch kits, and sushi; **side dishes and hors d’oeuvres** like plain pasta, noodles, rice, vegetable salads, potatoes, dumplings, and samosas; and **snack foods** such as chips, crackers, crispbread, and popcorn. See Table 1 for complete list of categories and details of the components of each category. ^†^The coefficient of variation (CV) for this estimate has high sampling variability (i.e., 16.6 > CV ≤ 33.3). ^‡^The coefficient of variation (CV) for this estimated has a very high coefficient of variation (i.e., CV > 33.3) and was therefore not released.
(DOCX)

S5 Table Top food categories contributing to calcium intakes of children at school, by age group, sex, and among all children, ranked by proportion contributed and including the mean amount per capita, the mean amount per consumer, and the number and proportion of children consuming each top category at school. 2015 CCHS-Nutrition – Public Use Microdata Files (n = 1,690). Note: The survey weights provided by Statistics Canada were applied to obtain nationally representative estimates. To account for the complex survey design, bootstrapping with 500 replicates was used to generate SE estimates. The counts (n’s) represent the unweighted number of children who consumed food in the category at school. Abbreviations: CCHS, Canadian Community Health Survey; NR, not reliable; SE, standard error. *Examples of foods in categories include: **Baked goods** such as muffins, cookies, granola bars, energy bars, protein bars, croissants, pastries, pies, cakes, and donuts; **combination dishes** including shepherd’s pie, chicken with rice and vegetables, beef and noodles, meat pies, vegetable and meat lasagna, macaroni and cheese, and vegetarian and meat chili; **handheld entrées** like sandwiches, wraps, burgers, pizza, hotdogs, lunch kits, and sushi; **fruits** including all fresh, frozen, cooked, bottled, canned, and dried fruit; and **snack foods** such as chips, crackers, crispbread, and popcorn. See Table 1 for complete list of categories and details of the components of each category. ^†^The coefficient of variation (CV) for this estimate has high sampling variability (i.e., 16.6 > CV ≤ 33.3). ^‡^The coefficient of variation (CV) for this estimated has a very high coefficient of variation (i.e., CV > 33.3) and was therefore not released.
(DOCX)

S6 Table Top food categories contributing to potassium intakes of children at school, by age group, sex, and among all children, ranked by proportion contributed and including the mean amount per capita, the mean amount per consumer, and the number and proportion of children consuming each top category at school. 2015 CCHS-Nutrition – Public Use Microdata Files (n = 1,690). Note: The survey weights provided by Statistics Canada were applied to obtain nationally representative estimates. To account for the complex survey design, bootstrapping with 500 replicates was used to generate SE estimates. The counts (n’s) represent the unweighted number of children who consumed food in the category at school. Abbreviations: CCHS, Canadian Community Health Survey; SE, standard error. *Examples of foods in categories include: **Baked goods** such as muffins, cookies, granola bars, energy bars, protein bars, croissants, pastries, pies, cakes, and donuts; **combination dishes** including shepherd’s pie, chicken with rice and vegetables, beef and noodles, meat pies, vegetable and meat lasagna, macaroni and cheese, and vegetarian and meat chili; **handheld entrées** like sandwiches, wraps, burgers, pizza, hotdogs, lunch kits, and sushi; **fruits** including all fresh, frozen, cooked, bottled, canned, and dried fruit; **snack foods** such as chips, crackers, crispbread, and popcorn; and **vegetables** including all fresh, frozen, cooked, bottled, canned, and dried vegetables. See Table 1 for complete list of categories and details of the components of each category. ^†^The coefficient of variation (CV) for this estimate has high sampling variability (i.e., 16.6 > CV ≤ 33.3).
(DOCX)

S7 Table Top food categories contributing to fibre intakes of children at school, by age group, sex, and among all children, ranked by proportion contributed and including the mean amount per capita, the mean amount per consumer, and the number and proportion of children consuming each top category at school. 2015 CCHS-Nutrition – Public Use Microdata Files (n = 1,690). Note: The survey weights provided by Statistics Canada were applied to obtain nationally representative estimates. To account for the complex survey design, bootstrapping with 500 replicates was used to generate SE estimates. The counts (n’s) represent the unweighted number of children who consumed food in the category at school. Abbreviations: CCHS, Canadian Community Health Survey; SE, standard error. *Examples of foods in categories include: **Baked goods** such as muffins, cookies, granola bars, energy bars, protein bars, croissants, pastries, pies, cakes, and donuts; **bread products** like breads, buns, bagels, biscuits, English muffins, pitas, tortillas, and bannock; **combination dishes** including shepherd’s pie, chicken with rice and vegetables, beef and noodles, meat pies, vegetable and meat lasagna, macaroni and cheese, and vegetarian and meat chili; **handheld entrées** like sandwiches, wraps, burgers, pizza, hotdogs, lunch kits, and sushi; **fruits** including all fresh, frozen, cooked, bottled, canned, and dried fruit; **side dishes and hors d’oeuvres** like plain pasta, noodles, rice, vegetable salads, potatoes, dumplings, and samosas; **snack foods** such as chips, crackers, crispbread, and popcorn; and **vegetables** including all fresh, frozen, cooked, bottled, canned, and dried vegetables. See Table 1 for complete list of categories and details of the components of each category. ^†^The coefficient of variation (CV) for this estimate has high sampling variability (i.e., 16.6 > CV ≤ 33.3).
(DOCX)

S8 Table Top food categories contributing to iron intakes of children at school, by age group, sex, and among all children, ranked by proportion contributed and including the mean amount per capita, the mean amount per consumer, and the number and proportion of children consuming each top category at school. 2015 CCHS-Nutrition – Public Use Microdata Files (n = 1,690). Note: The survey weights provided by Statistics Canada were applied to obtain nationally representative estimates. To account for the complex survey design, bootstrapping with 500 replicates was used to generate SE estimates. The counts (n’s) represent the unweighted number of children who consumed food in the category at school. Abbreviations: CCHS, Canadian Community Health Survey; SE, standard error. *Examples of foods in categories include: **Baked goods** such as muffins, cookies, granola bars, energy bars, protein bars, croissants, pastries, pies, cakes, and donuts; **bread products** like breads, buns, bagels, biscuits, English muffins, pitas, tortillas, and bannock; **combination dishes** including shepherd’s pie, chicken with rice and vegetables, beef and noodles, meat pies, vegetable and meat lasagna, macaroni and cheese, and vegetarian and meat chili; **handheld entrées** like sandwiches, wraps, burgers, pizza, hotdogs, lunch kits, and sushi; **fruits** including all fresh, frozen, cooked, bottled, canned, and dried fruit; **side dishes and hors d’oeuvres** like plain pasta, noodles, rice, vegetable salads, potatoes, dumplings, and samosas; **snack foods** such as chips, crackers, crispbread, and popcorn; and **vegetables** including all fresh, frozen, cooked, bottled, canned, and dried vegetables. See Table 1 for complete list of categories and details of the components of each category. ^†^The coefficient of variation (CV) for this estimate has high sampling variability (i.e., 16.6 > CV ≤ 33.3).
(DOCX)

S9 Table Top food categories contributing to vitamin A intakes of children at school, by age group, sex, and among all children, ranked by proportion contributed and including the mean amount per capita, the mean amount per consumer, and the number and proportion of children consuming each top category at school. 2015 CCHS-Nutrition – Public Use Microdata Files (n = 1,690). Note: The survey weights provided by Statistics Canada were applied to obtain nationally representative estimates. To account for the complex survey design, bootstrapping with 500 replicates was used to generate SE estimates. The counts (n’s) represent the unweighted number of children who consumed food in the category at school. Abbreviations: CCHS, Canadian Community Health Survey; RAE, Retinol Activity Equivalents; SE, standard error. *Examples of foods in categories include: **Baked goods** such as muffins, cookies, granola bars, energy bars, protein bars, croissants, pastries, pies, cakes, and donuts; **combination dishes** including shepherd’s pie, chicken with rice and vegetables, beef and noodles, meat pies, vegetable and meat lasagna, macaroni and cheese, and vegetarian and meat chili; **handheld entrées** like sandwiches, wraps, burgers, pizza, hotdogs, lunch kits, and sushi; **fruits** including all fresh, frozen, cooked, bottled, canned, and dried fruit; **side dishes and hors d’oeuvres** like plain pasta, noodles, rice, vegetable salads, potatoes, dumplings, and samosas; and **vegetables** including all fresh, frozen, cooked, bottled, canned, and dried vegetables. See Table 1 for complete list of categories and details of the components of each category. ^†^The coefficient of variation (CV) for this estimate has high sampling variability (i.e., 16.6 > CV ≤ 33.3).
(DOCX)

## Abbreviations

24HR: 24-hour dietary recall
CCHS: Canadian Community Health Survey.
