# Supplementary material for: Top food categories contributing to Canadian children’s energy and nutrient intakes at school
Source: PLoS One. 2026 Jan 13;21(1):e0340494. doi: 10.1371/journal.pone.0340494 (PMC12798986; doi:10.1371/journal.pone.0340494)
Supplement: S1 Table — 2015 CCHS-Nutrition – Public Use Microdata Files (n = 1,690). Note: The survey weights provided by Statistics Canada were applied to obtain nationally representative estimates. To account for the complex survey design, bootstrapping with 500 replicates was used to generate SE estimates. The counts (n’s) represent the unweighted number of children who consumed food in the category at school. Abbreviations: CCHS, Canadian Community Health Survey; SE, standard error. *Examples of foods in categories include: Baked goods such as muffins, cookies, granola bars, energy bars, protein bars, croissants, pastries, pies, cakes, and donuts; bread products like breads, buns, bagels, biscuits, English muffins, pitas, tortillas, and bannock; combination dishes including shepherd’s pie, chicken with rice and vegetables, beef and noodles, meat pies, vegetable and meat lasagna, macaroni and cheese, and vegetarian and meat chili; handheld entrées like sandwiches, wraps, burgers, pizza, hotdogs, lunch kits, and sushi; fruits including all fresh, frozen, cooked, bottled, canned, and dried fruit; side dishes and hors d’oeuvres like plain pasta, noodles, rice, vegetable salads, potatoes, dumplings, and samosas; and snack foods such as chips, crackers, crispbread, and popcorn. See Table 1 for complete list of categories and details of the components of each category. †The coefficient of variation (CV) for this estimate has high sampling variability (i.e., 16.6 > CV ≤ 33.3). (DOCX) [file pone.0340494.s001.docx]

**Table S1**. Top food categories contributing to energy intakes of children at school, by age group, sex, and among all children, ranked by proportion contributed and including the mean amount per capita, the mean amount per consumer, and the number and proportion of children consuming each top category at school. 2015 CCHS-Nutrition – Public Use Microdata Files (n=1,690).

|  | Food categories* | % contributed | mean (SE) amount per capita (kcal) | mean (SE) amount per consumer (kcal) | n (%) individuals consuming category |
| --- | --- | --- | --- | --- | --- |
| Overall | | | | | |
| All children (n=1,690) | 1. Handheld entrées | 25.6 | 157 (13) | 351 (19) | 780 (44.7) |
|  | 2. Baked goods | 13.7 | 84 (5) | 186 (7) | 784 (45.4) |
|  | 3. Combination dishes | 9.7 | 60 (7) | 388 (26) | 203 (15.4) |
|  | 4. Fruits | 8.4 | 52 (2) | 94 (3) | 859 (55.2) |
|  | 5. Snack foods | 8.2 | 50 (6) | 177 (18) | 491 (28.4) |
|  | 6. Fruit and vegetable juice and drinks | 6.6 | 40 (3) | 113 (6) | 533 (35.7) |
|  | 7. Meat and poultry | 4.4 | 27 (4) | 211 (19) | 176 (12.9) |
|  | 8. Milk, yogurt drinks, and plant-based beverages | 3.9 | 24 (3) | 142 (9) | 293 (16.8) |
|  | 9. Bread products | 3.1 | 19 (3) | 170 (12) | 182 (11.2) |
|  | 10. Side dishes and hors d'oeuvres | 3.1^†^ | 19 (3)^†^ | 177 (19) | 171 (10.6) ^†^ |
| Age Group | | | | | |
| Younger children (i.e., 4-9 y; n=575) | 1. Handheld entrées | 22.7 | 134 (13) | 286 (14) | 282 (46.8) |
|  | 2. Baked goods | 14.5 | 86 (8) | 169 (9) | 308 (50.6) |
|  | 3. Fruits | 10.5 | 62 (4) | 92 (4) | 357 (67.7) |
| Adolescents (i.e., 10-18 y; n=1,115) | 1. Handheld entrées | 27.8 | 175 (19) | 407 (32) | 498 (42.9) |
|  | 2. Baked goods | 13.2 | 83 (6) | 202 (12) | 476 (41.3) |
|  | 3. Combination dishes | 11.2^†^ | 71 (9) | 432 (37) | 140 (16.4) |
| Sex | | | | | |
| Males (n=842) | 1. Handheld entrées | 27.4 | 175 (14) | 360 (18) | 430 (48.7) |
|  | 2. Baked goods | 16.1 | 103 (8) | 202 (12) | 404 (51.3) |
|  | 3. Combination dishes | 9.3^†^ | 60^†^ (10) | 433 (51) | 88 ^†^ (13.8) |
| Females (n=848) | 1. Handheld entrées | 23.6 | 139 (14) | 341 (40) | 350 (40.6) |
|  | 2. Baked goods | 11.1 | 65 (8) | 165 (8) | 380 (39.5) |
|  | 3. Combination dishes | 10.2 | 60^†^ (10) | 351 (24) | 115 (17.0) |

Note: The survey weights provided by Statistics Canada were applied to obtain nationally representative estimates. To account for the complex survey design, bootstrapping was used to generate SE estimates. The counts (n’s) represent the unweighted number of children who consumed food in the category at school.

Abbreviations: CCHS, Canadian Community Health Survey; SE, standard error.

*Examples of foods in categories include: **Baked goods** such as muffins, cookies, granola bars, energy bars, protein bars, croissants, pastries, pies, cakes, and donuts; **bread products** like breads, buns, bagels, biscuits, English muffins, pitas, tortillas, and bannock; **combination dishes** including shepherd’s pie, chicken with rice and vegetables, beef and noodles, meat pies, vegetable and meat lasagna, macaroni and cheese, and vegetarian and meat chili; **handheld entrées** like sandwiches, wraps, burgers, pizza, hotdogs, lunch kits, and sushi; **fruits** including all fresh, frozen, cooked, bottled, canned, and dried fruit; **side dishes and hors d'oeuvres** like plain pasta, noodles, rice, vegetable salads, potatoes, dumplings, and samosas; and **snack foods** such as chips, crackers, crispbread, and popcorn. See Table 1 for complete list of categories and details of the components of each category.

^†^The coefficient of variation (CV) for this estimate has high sampling variability (i.e., 16.6> CV ≤33.3).
