# Supplementary material for: Top food categories contributing to Canadian children’s energy and nutrient intakes at school
Source: PLoS One. 2026 Jan 13;21(1):e0340494. doi: 10.1371/journal.pone.0340494 (PMC12798986; doi:10.1371/journal.pone.0340494)
Supplement: S3 Table — 2015 CCHS-Nutrition – Public Use Microdata Files (n = 1,690). Note: The survey weights provided by Statistics Canada were applied to obtain nationally representative estimates. To account for the complex survey design, bootstrapping with 500 replicates was used to generate SE estimates. The counts (n’s) represent the unweighted number of children who consumed food in the category at school. Abbreviations: CCHS, Canadian Community Health Survey; SE, standard error. *Examples of foods in categories include: Baked goods such as muffins, cookies, granola bars, energy bars, protein bars, croissants, pastries, pies, cakes, and donuts; combination dishes including shepherd’s pie, chicken with rice and vegetables, beef and noodles, meat pies, vegetable and meat lasagna, macaroni and cheese, and vegetarian and meat chili; handheld entrées like sandwiches, wraps, burgers, pizza, hotdogs, lunch kits, and sushi; other beverages such as soda, energy drinks, sports drinks, vitamin water, lemonade, and flavored water; and fruits including all fresh, frozen, cooked, bottled, canned, and dried fruit. See Table 1 for complete list of categories and details of the components of each category. †The coefficient of variation (CV) for this estimate has high sampling variability (i.e., 16.6 > CV ≤ 33.3). (DOCX) [file pone.0340494.s003.docx]

**Table S3**. Top food categories contributing to sugar intakes of children at school, by age group, sex, and among all children, ranked by proportion contributed and including the mean amount per capita, the mean amount per consumer, and the number and proportion of children consuming each top category at school. 2015 CCHS-Nutrition – Public Use Microdata Files (n=1,690).

|  | Food categories* | % contributed | mean (SE) amount per capita (g) | mean (SE) amount per consumer (g) | n (%) individuals consuming category |
| --- | --- | --- | --- | --- | --- |
| Overall | | | | | |
| All children (n=1,690) | 1. Fruits | 24.7 | 9.6 (0.4) | 17.4 (0.5) | 859 (55.2) |
|  | 2. Fruit and vegetable juice and drinks | 20.9 | 8.1 (0.6) | 22.8 (1.4) | 533 (35.7) |
|  | 3. Baked goods | 15.7 | 6.1 (0.4) | 13.5 (0.6) | 784 (45.4) |
|  | 4. Milk, yogurt drinks, and plant-based beverages | 7.8 | 3.0 (0.3) | 18.1 (1.1) | 293 (16.8) |
|  | 5. Handheld entrées | 7.7^†^ | 3.0^†^ (0.8) | 6.7^†^ (1.8) | 780 (44.7) |
|  | 6. Candies, chocolate, and desserts | 5.6^†^ | 2.2^†^ (0.4) | 19.9 (2.7) | 220 (10.9) |
|  | 7. Yogurt | 3.9 | 1.5 (0.2) | 9.6 (0.6) | 244 (15.6) |
|  | 8. Other beverages | 3.0^†^ | 1.2^†^ (0.3) | 19.2 (2.4) | 121 (6.1^†^) |
|  | 9. Combination dishes | 2.0^†^ | 0.8 (0.1) | 5.0 (0.5) | 203 (15.4) |
|  | 10. Coffee, tea, and hot chocolate | 1.6^†^ | 0.6^†^ (0.2) | 16.0^†^ (2.7) | 61 (4.0^†^) |
| Age Group | | | | | |
| Younger children (i.e., 4-9 y; n=575) | 1. Fruits | 28.3 | 11.5 (0.8) | 17 (0.8) | 357 (67.7) |
|  | 2. Fruit and vegetable juice and drinks | 20.6 | 8.4 (0.7) | 19.2 (0.7) | 213 (43.6) |
|  | 3. Baked goods | 14.7 | 6.0 (0.6) | 11.8 (0.9) | 308 (50.6) |
| Adolescents (i.e., 10-18 y; n=1,115) | 1. Fruits | 21.5 | 8.1 (0.5) | 17.8 (0.6) | 502 (45.2) |
|  | 2. Fruit and vegetable juice and drinks | 21.2 | 7.9 (1.0) | 26.9 (2.6) | 320 (29.4) |
|  | 3. Baked goods | 16.6 | 6.2 (0.5) | 15.1 (1.0) | 476 (41.3) |
| Sex | | | | | |
| Males (n=842) | 1. Fruits | 23.7 | 9.5 (0.6) | 17.6 (0.6) | 397 (54.0) |
|  | 2. Fruit and vegetable juice and drinks | 19.8 | 8.0 (0.8) | 22.4 (1.4) | 274 (35.5) |
|  | 3. Baked goods | 18.7 | 7.5 (0.7) | 14.7 (1.1) | 404 (51.3) |
| Females (n=848) | 1. Fruits | 25.7 | 9.6 (0.6) | 17.1 (0.8) | 462 (56.3) |
|  | 2. Fruit and vegetable juice and drinks | 22.1 | 8.3 (0.8) | 23.1 (2.4) | 259 (35.9) |
|  | 3. Baked goods | 12.5 | 4.7 (0.7) | 11.9 (0.7) | 380 (39.5) |

Note: The survey weights provided by Statistics Canada were applied to obtain nationally representative estimates. To account for the complex survey design, bootstrapping with 500 replicates was used to generate SE estimates. The counts (n’s) represent the unweighted number of children who consumed food in the category at school.

Abbreviations: CCHS, Canadian Community Health Survey; SE, standard error.

*Examples of foods in categories include: **Baked goods** such as muffins, cookies, granola bars, energy bars, protein bars, croissants, pastries, pies, cakes, and donuts; **combination dishes** including shepherd’s pie, chicken with rice and vegetables, beef and noodles, meat pies, vegetable and meat lasagna, macaroni and cheese, and vegetarian and meat chili; **handheld entrées** like sandwiches, wraps, burgers, pizza, hotdogs, lunch kits, and sushi; **other beverages** such as soda, energy drinks, sports drinks, vitamin water, lemonade, and flavored water; and **fruits** including all fresh, frozen, cooked, bottled, canned, and dried fruit. See Table 1 for complete list of categories and details of the components of each category.

^†^The coefficient of variation (CV) for this estimate has high sampling variability (i.e., 16.6> CV ≤33.3).
