# Supplementary material for: Top food categories contributing to Canadian children’s energy and nutrient intakes at school
Source: PLoS One. 2026 Jan 13;21(1):e0340494. doi: 10.1371/journal.pone.0340494 (PMC12798986; doi:10.1371/journal.pone.0340494)
Supplement: S5 Table — 2015 CCHS-Nutrition – Public Use Microdata Files (n = 1,690). Note: The survey weights provided by Statistics Canada were applied to obtain nationally representative estimates. To account for the complex survey design, bootstrapping with 500 replicates was used to generate SE estimates. The counts (n’s) represent the unweighted number of children who consumed food in the category at school. Abbreviations: CCHS, Canadian Community Health Survey; NR, not reliable; SE, standard error. *Examples of foods in categories include: Baked goods such as muffins, cookies, granola bars, energy bars, protein bars, croissants, pastries, pies, cakes, and donuts; combination dishes including shepherd’s pie, chicken with rice and vegetables, beef and noodles, meat pies, vegetable and meat lasagna, macaroni and cheese, and vegetarian and meat chili; handheld entrées like sandwiches, wraps, burgers, pizza, hotdogs, lunch kits, and sushi; fruits including all fresh, frozen, cooked, bottled, canned, and dried fruit; and snack foods such as chips, crackers, crispbread, and popcorn. See Table 1 for complete list of categories and details of the components of each category. †The coefficient of variation (CV) for this estimate has high sampling variability (i.e., 16.6 > CV ≤ 33.3). ‡The coefficient of variation (CV) for this estimated has a very high coefficient of variation (i.e., CV > 33.3) and was therefore not released. (DOCX) [file pone.0340494.s005.docx]

**Table S5**. Top food categories contributing to calcium intakes of children at school, by age group, sex, and among all children, ranked by proportion contributed and including the mean amount per capita, the mean amount per consumer, and the number and proportion of children consuming each top category at school. 2015 CCHS-Nutrition – Public Use Microdata Files (n=1,690).

|  | Food categories* | % contributed | mean (SE) amount per capita (mg) | mean (SE) amount per consumer (mg) | n (%) individuals consuming category |
| --- | --- | --- | --- | --- | --- |
| Overall | | | | | |
| All children (n=1,690) | 1. Handheld entrées | 26.5 | 71 (6) | 160 (9) | 780 (44.7) |
|  | 2. Milk, yogurt drinks, and plant-based beverages | 16.4 | 44 (4) | 264 (16) | 293 (16.8) |
|  | 3. Cheese | 12.5 | 34 (4) | 192 (16) | 256 (17.5) |
|  | 4. Baked goods | 7.1 | 19 (2) | 42 (3) | 784 (45.4) |
|  | 5. Yogurt | 6.0 | 16 (2) | 104 (5) | 244 (15.6) |
|  | 6. Fruit and vegetable juice and drinks | 5.3^†^ | 14^†^ (5) | NR^‡^ | 533 (35.7) |
|  | 7. Combination dishes | 5.0 | 13 (2) | 88 (14) | 203 (15.4) |
|  | 8. Fruits | 3.7 | 10 (1) | 18 (1) | 859 (55.2) |
|  | 9. Snack foods | 3.1 | 8 (1) | 29 (2) | 491 (28.4) |
|  | 10. Water | 2.8 | 8 (0) | 12 (1) | 1014 (64.8) |
| Age Group | | | | | |
| Younger children (i.e., 4-9 y; n=575) | 1. Milk, yogurt drinks, and plant-based beverages | 22.2^†^ | 64 (8) | 261 (22) | 133 (24.6) |
|  | 2. Handheld entrées | 20.5 | 59 (6) | 127 (9) | 282 (46.8) |
|  | 3. Cheese | 17.4 | 50 (8) | 194 (22) | 135 (26.0) |
| Adolescents (i.e., 10-18 y; n=1,115) | 1. Handheld entrées | 31.9 | 81 (9) | 188 (15) | 498 (42.9) |
|  | 2. Milk, yogurt drinks, and plant-based beverages | 11.2 | 28 (4) | 269 (24) | 160 (10.5) |
|  | 3. Baked goods | 8.4 | 21 (2) | 52 (5) | 476 (41.3) |
| Sex | | | | | |
| Males (n=842) | 1. Handheld entrées | 29.9 | 77 (8) | 159 (11) | 430 (48.7) |
|  | 2. Milk, yogurt drinks, and plant-based beverages | 14.8 | 38 (6) | 238 (15) | 159 (16.0) |
|  | 3. Cheese | 12.1 | 31 (5) | 196 (22) | 123 (15.9) |
| Females (n=848) | 1. Handheld entrées | 23.3 | 65 (8) | 161 (17) | 350 (40.6) |
|  | 2. Milk, yogurt drinks, and plant-based beverages | 18.0^†^ | 50 (6) | 287 (29) | 134 (17.5) |
|  | 3. Cheese | 12.9^†^ | 36 (5) | 189 (23) | 133 (19.1) |

Note: The survey weights provided by Statistics Canada were applied to obtain nationally representative estimates. To account for the complex survey design, bootstrapping with 500 replicates was used to generate SE estimates. The counts (n’s) represent the unweighted number of children who consumed food in the category at school.

Abbreviations: CCHS, Canadian Community Health Survey; NR, not reliable; SE, standard error.

*Examples of foods in categories include: **Baked goods** such as muffins, cookies, granola bars, energy bars, protein bars, croissants, pastries, pies, cakes, and donuts; **combination dishes** including shepherd’s pie, chicken with rice and vegetables, beef and noodles, meat pies, vegetable and meat lasagna, macaroni and cheese, and vegetarian and meat chili; **handheld entrées** like sandwiches, wraps, burgers, pizza, hotdogs, lunch kits, and sushi; **fruits** including all fresh, frozen, cooked, bottled, canned, and dried fruit; and **snack foods** such as chips, crackers, crispbread, and popcorn. See Table 1 for complete list of categories and details of the components of each category.

^†^The coefficient of variation (CV) for this estimate has high sampling variability (i.e., 16.6> CV ≤33.3).

^‡^The coefficient of variation (CV) for this estimated has a very high coefficient of variation (i.e., CV>33.3) and was therefore not released.
